# Biomarkers of neuronal damage in saturation diving—a controlled observational study

**DOI:** 10.1007/s00421-020-04499-y

**Published:** 2020-09-25

**Authors:** Anders Rosén, Mikael Gennser, Nicklas Oscarsson, Andreas Kvarnström, Göran Sandström, Kaj Blennow, Helen Seeman-Lodding, Henrik Zetterberg

**Affiliations:** 1 Department of Anesthesia and Intensive Care Medicine, Sahlgrenska University Hospital, Gothenburg, Sweden; 2 Department of Environmental Physiology, School of Chemistry, Biotechnology and Health, Royal Institute of Technology, KTH, Stockholm, Sweden; 3 Department of Anesthesia and Intensive Care Medicine, Institute of Clinical Sciences, Sahlgrenska Academy, University of Gothenburg, Gothenburg, Sweden; 4 Swedish Armed Forces, Center for Defence Medicine, Gothenburg, Sweden; 5 Clinical Neurochemistry Laboratory, Sahlgrenska University Hospital, Gothenburg, Sweden; 6 Department of Psychiatry and Neurochemistry, Sahlgrenska Academy, University of Gothenburg, Gothenburg, Sweden; 7 Department of Neurodegenerative Disease, University College London, UCL, Institute of Neurology, London, United Kingdom; 8 UK Dementia Research Institute at University College London, UCL, London, United Kingdom

**Keywords:** Biomarkers, Central nervous system, Diving, hyperbaric, Neuronal damage, Saturation diving

## Abstract

**Purpose:**

A prospective and controlled observational study was performed to determine if the central nervous system injury markers glial fibrillary acidic protein (GFAp), neurofilament light (NfL) and tau concentrations changed in response to a saturation dive.

**Methods:**

The intervention group consisted of 14 submariners compressed to 401 kPa in a dry hyperbaric chamber. They remained pressurized for 36 h and were then decompressed over 70 h. A control group of 12 individuals was used. Blood samples were obtained from both groups before, during and after hyperbaric exposure, and from the intervention group after a further 25–26 h.

**Results:**

There were no statistically significant changes in the concentrations of GFAp, NfL and tau in the intervention group. During hyperbaric exposure, GFAp decreased in the control group (mean/median − 15.1/ − 8.9 pg·mL^−1^, *p* < 0.01) and there was a significant difference in absolute change of GFAp and NfL between the groups (17.7 pg·mL^−1^, *p* = 0.02 and 2.34 pg·mL^−1^, *p* = 0.02, respectively). Albumin decreased in the control group (mean/median − 2.74 g/L/ − 0.95 g/L, *p* = 0.02), but there was no statistically significant difference in albumin levels between the groups. In the intervention group, haematocrit and mean haemoglobin values were slightly increased after hyperbaric exposure (mean/median 2.3%/1.5%, *p* = 0.02 and 4.9 g/L, *p* = 0.06, respectively).

**Conclusion:**

Hyperbaric exposure to 401 kPa for 36 h was not associated with significant increases in GFAp, NfL or tau concentrations. Albumin levels, changes in hydration or diurnal variation were unlikely to have confounded the results. Saturation exposure to 401 kPa seems to be a procedure not harmful to the central nervous system.

**Trial registration:**

ClinicalTrials.gov NCT03192930.

## Introduction

Divers are repeatedly exposed to increased ambient pressures and both caisson and tunnel workers might have to work in a compressed air environment, typically exposed to 1.5–7 times normal atmospheric pressure (Le Péchon 2010). The hyperbaric environment is potentially dangerous to the human body and adverse effects can manifest both during and after exposure.

Divers are often exposed to increased partial pressures of oxygen. By maintaining the oxygen fraction in the breathing gas above what would constitute a normoxic partial pressure, the uptake of inert gas is reduced and the time needed for decompression reduced. However, increased partial pressures of oxygen have toxic effects, mainly affecting the lungs and the central nervous system. The toxic effect of oxygen on the central nervous system (CNS) depends mainly on its partial pressure, time of exposure, and individual susceptibility. Toxicity could be aggravated by exercise, hypothermia, immersion in water or hypercarbia (Edmonds 2016a). Oxygen toxicity becomes increasingly noticeable at partial pressures exceeding 150 kPa, equivalent to a depth of about 60 m of seawater (msw) when breathing air (Hamilton 1989). Symptoms of oxygen toxicity are facial twitching, nausea, dizziness, tinnitus, tunnel vision and generalized seizures (Edmonds 2016b).

Nitrogen affects the nervous system when its partial pressure increases, which typically manifests as changed behaviour, impaired intellectual performance, and deteriorating neuromuscular function. The symptoms usually become apparent as the nitrogen pressure exceeds 300 kPa, equivalent to a depth of about 30msw when breathing air, and gradually worsen with increasing pressure. Factors, such as compression rate and level of carbon dioxide as well as anxiety and stress, accentuate the effects (Bennet 2003).

While diving using air as breathing gas, nitrogen will accumulate in the body due to the increased ambient pressure. Deep, extended or repeated diving could result in considerable uptake of nitrogen. When the diver returns towards the surface and ambient pressure decreases, nitrogen will leave the tissues and enter the blood before finally being exhaled through the lungs. At this stage, supersaturation can lead to formation of inert gas bubbles in both blood and tissues, which is common after diving. Intravascular inert gas bubbles form mainly in the venous system and are referred to as venous gas emboli (VGE) (Francis 2003). The amount of VGE is positively correlated with decompression speed (Germonpré 2017), but there is a considerable individual variability in the amount of VGEs observed in divers after similar decompression regimens (Papadopoulou 2018). Inert gas bubbles are considered to be the cause of decompression sickness (DCS). Analyses of large groups of divers show that DCS is more common when the VGE load is high. (Nishi 2003, Eftedal 2007). Still, divers may exhibit a high VGE load without any signs of DCS and divers without detectable VGE can develop DCS.

Saturation diving is a technique developed to handle the risk of DCS in conjunction with prolonged or deep dives. In contrast to usual diving, where each session begins and ends at the surface, in saturation diving, the divers remain under pressure for long periods of time, usually days to weeks. The inert gas pressures in the divers’ tissues are, during this period, in equilibrium with the ambient inert gas pressure and they are, hence, saturated (Powell 2014).

Though neurological impairment is evident during exposure to increased partial pressures of nitrogen and oxygen, and in connection to DCS involving the central nervous system, possible neurological long-term effects of uneventful diving remain to be fully elucidated (Grønning and Aarli 2011). Experienced saturation divers have, when compared to non-diving controls, been found to have more subjectively reported problems with memory and concentration, as well as more neurological symptoms when objectively examined (Todnem 1990,1991), but confounding factors (e.g., DCS) exist and causality between hyperbaric exposure per se and neuropsychological sequelae remains to be unequivocally proven. There are reports of objectively confirmed impaired memory function among both saturation divers with subjective forgetfulness (Taylor 2006) and experienced recreational divers without a history of DCS (Hemelryck 2014). Assessment of cognitive function using neuropsychological tests showed a correlation between frequent recreational diving in cold water and decreased cognitive performance (Slosman 2004) and worse visual–motor performance and short-term memory has been reported among recreational divers (Balestra 2016). In contrast, another study comparing professional divers to matched controls found no difference in neuropsychometric test results (Cordes 2000), and a 12-year longitudinal study on professional non-saturation divers concluded that divers without a history of DCS did not show signs of impaired nervous system function (Bast-Petrersen 2015).

It is unknown to what extent factors, such as increased hydrostatic pressure or higher than normal oxygen and nitrogen partial pressures, influence neurons in the CNS. If there is a cumulative effect on the CNS due to multiple exposures to a hyperbaric environment, it should be possible to measure acute effects after provocative dives, even if those effects are subclinical and do not give rise to any subjective symptoms.

We speculated that a saturation dive would infer neuronal stress and possibly be harmful to the CNS, which eventually could translate into long-term neurological sequelae. To test the hypothesis that a saturation dive might cause injury to the CNS, we selected a number of CNS-specific molecules in blood, Glial fibrillary acidic protein (GFAp), Neurofilament light protein (NfL) and tau protein (tau), the concentrations of which are known to increase in response to different types of brain trauma, including mild traumatic brain injury (Zetterberg 2016), and neuronal stress (Evered 2018, Sato 2018). Our aim was to analyse the concentrations of these proteins in blood among individuals exposed to an increased ambient pressure and compare the results with those obtained in unexposed individuals.

GFAp has a molecular weight of 50 kDa and is expressed mainly in astrocytes in the CNS. Elevated levels of GFAp in blood have been reported after traumatic brain injury (Zetterberg 2016, Gill 2018) and after intracerebral haemorrhage (Foerch 2012). However, a change in GFAp concentration is also claimed to reflect astrocytic plasticity in response to neuronal stress and increased neuronal metabolic and immunologic activity (Wang 2009, Brenner 2014, Femenia 2018).

NfL is a structural axonal protein, with a molecular weight of 68 kDa, found mainly in myelinated subcortical axons. NfL levels in blood are increased in patients with traumatic brain injury (Shahim 2016, Zetterberg 2016), axonal injury due to multiple sclerosis (Khalil 2018), and sports-related concussion (Shahim 2018), but even uneventful anaesthesia has been associated with increased blood levels of NfL (Evered 2018).

Tau is a microtubule-binding protein present in neuronal axons, mainly in the cortex, with a molecular weight of 55–62 kDa, which is thought to be a marker of both axonal damage and neuronal plasticity in response to stress. Tau could be both passively released as a result of cell death but also actively secreted in connection to increased neuronal activity (Sato 2018). Increased levels of tau have been reported in the context of dementia, brain injury (Zetterberg 2016, Mattson 2017), cerebral concussion (Shahim 2014, Zetterberg 2016) and boxing (Neselius 2012, Zetterberg 2006), but also after less evident neuronal trauma as protracted apnea (Gren 2016) and uneventful anaesthesia (Evered 2018). A small pilot study found elevated tau levels in blood after diving (Rosén 2019). However, another small pilot study found no increase in tau levels in cerebrospinal fluid among divers with DCS (Shahim 2015).

We had the possibility to analyse GFAp, NfL, and tau using a 4-plex Single molecule (Simoa) assay, in which also ubiquitin carboxy-terminal hydrolase L1 (UCH-L1) was included. This is a cytoplasmic neuronal protein present in the CNS, the peripheral nervous system, the neuroendocrine system, endothelial and smooth muscle cells. Increased concentrations of UCH-L1 have been reported in patients with traumatic brain injury but the analytical performance of the biomarker in the 4-plex assay is variable (Zetterberg 2016).

## Materials and methods

The study was prospective, controlled and observational. It was conducted in accordance with the Declaration of Helsinki, approved by the regional ethical committee in Gothenburg, Sweden (Dnr 022-17), registered at ClinicalTrials.gov (NCT03190252) and carried out at the naval base in Karlskrona, Sweden, as two separate but identical trials, between March 17th and 23rd 2017, and between January 11th and 17th 2018, while the Swedish armed forces (SwAF) performed validation tests of a decompression table to be used during submarine rescue. Written consent was obtained from all subjects before participation in the study.

The intervention group consisted of 14 Swedish Navy submariners participating in the SwAF submarine rescue training. The control group consisted of 12 people who either had passed a dive medical examination or were employed as Swedish Navy mariners. None of the control subjects undertook any diving activities during the study.

Data concerning gender, age, weight, height and current medication were collected from all subjects and baseline venous blood samples taken (sample 1, between 09:20 and 14:00). The intervention groups were compressed in a dry hyperbaric chamber to 401 kPa, equivalent to a depth of 30msw, and remained at that pressure for 36 h. They were then decompressed at a rate of 0.5 m/h for 30 h, thereafter 0.375 m/h for 40 h. After 70 h of decompression, normal atmospheric pressure was reached and the subjects left the hyperbaric chamber, at which point they had then been subject to an increased ambient pressure for 106 h. The oxygen partial pressure inside the chamber was kept at or near 50 kPa at depth, which was accomplished by increasing the amount of nitrogen in the breathing gas. During decompression, the oxygen partial pressure was maintained at 50 kPa until 15msw (251 kPa) was reached. At that pressure, the breathing gas was switched to air and the decompression speed was reduced. During night hours, lights in the hyperbaric chamber were turned off and subjects in the intervention group lay in their beds. Some subjects in the control group were part of the ships rota of duties including night service. The start of compression of the intervention group was defined as 0 h. Venous blood samples were obtained from all subjects in both groups at 33–34 h (sample 2, between 06:10 and 08:00) and at 104–108 h (sample 3, between 05:30 and 09:40). For practical reasons, some samples in the control group had to be obtained shortly before the hyperbaric exposure ended at 106 h. Blood samples taken from the intervention group at 33–34 h were collected by a research nurse who was pressurized to 401 kPa and after sampling decompressed to atmospheric pressure. Sample 3 was obtained from the intervention group directly after the hyperbaric exposure had ended. About 25–26 h after the hyperbaric exposure ended, a venous blood sample was collected from subjects in the intervention group (sample 4, between 08:00 and 09:10). At 105 h, when the intervention group reached a pressure equivalent to 3msw (131 kPa), research personnel entered the chamber to monitor the subjects for VGE using precordial Doppler ultrasound (DBM9008, Techno Scientific Inc, Ontario, Canada). After the final decompression, repeated precordial Doppler ultrasound examinations were carried out until more than 3 h had elapsed.

All samples were analysed for GFAp, NfL, tau, UCH-L1 and albumin. They were collected in gel tubes (Vacuette no. 454420, Hettish Labinstrument AB, Sweden) and centrifuged for 10 min at 2200 rpm and 20° centigrade (Sorvall ST 8 / 8R Centrifuge, Thermo Scientific, Germany). Directly afterwards, aliquots of 500 μL serum were frozen on dry ice and then stored at − 78° centigrade until analyzed. GFAp, NfL, tau and UCH-L1 concentrations were measured using the Human Neurology 4-Plex A assay on an HD-1 Simoa instrument according to instructions from the manufacturer (Quanterix, Billerica, MA, USA).

For quality control (QC) samples with GFAp concentrations of 113.1 pg·mL^−1^ and 88.8 pg·mL^−1^, coefficients of variation (CVs) were 4.4% and 4.2%, respectively, for QC samples with NfL concentrations of 13.8 pg·mL^−1^ and 7.5 pg·mL^−1^, CVs were 4.6% and 5.7% and for quality control (QC) samples with tau concentrations of 1.5 pg·mL^−1^ and 23.9 pg·mL^−1^, CVs were 9.0% and 6.2%, respectively. The results of UCH-L1 analyses were discarded due to an unacceptably high level of imprecision as CVs were 105.6% and 21.7% for QC samples with UCH-L1 concentrations of 2.8 pg·mL^−1^ and 11.0 pg·mL^−1^, respectively.

Albumin concentration was measured using an immunoturbidimetric method on Elecsys (Roche Diagnostics, Penzberg, Germany).

In the intervention group, samples 1–3 were taken in doublets and each extra sample was analysed using a hand-held blood analyser (i-STAT^®^ 1, Abbott Point of Care Inc, IL, USA), which determined haematocrit (Hct) conductometrically and provided a calculated haemoglobin (Hb) value based on the Hct value. All samples for Hct and Hb taken during hyperbaric exposure (sample 2) had to be discarded, as potential VGE induced by decompression of the samples interfered with the results of the measured conductivity.

### Statistics

Statistical results for GFAp, NfL and tau were compiled using SAS^®^ v9.3 (Cary, NC, USA) in collaboration with an independent statistical company (Statistiska Konsultgruppen, Gothenburg, Sweden). Concentrations of GFAp, NfL and tau were presented as both mean (± standard deviation) and median (min;max) values. Differences between baseline and sample 2–4 were presented as absolute (pg·mL^−1^) changes. Fisher’s non-parametric permutation test was used for analyses of absolute changes within groups as well as for comparison of absolute changes between the two groups.

Statistical analyses for Hb, Hct and albumin were performed using IBM SPSS^®^ v24 (IBM, Armonk, NY, USA). Concentrations of albumin, Hb and Hct were presented as both mean (± standard deviation) and median (min;max) values. Within each group, absolute values of sample 2–4 were compared to baseline values using Wilcoxon signed-rank test. For comparison of albumin values between groups, Mann–Whitney *U* test was used.

In all comparisons, a two-sided *p* value of 0.05 or lower was considered statistically significant.

### Missing data

There were no missing data for GFAp, NfL or tau. In the intervention group, data on Hct were missing for one subject at baseline due to sampling error, on albumin for two subjects in sample 2, and for one subject in sample 3. In the control group, data on albumin were missing for two subjects at baseline and for one subject in samples 2 and 3. All missing data on albumin were caused by lack of sample volume.

## Results

### Demographics

There were significant differences in body mass index (BMI) and age between subjects in the two groups. Subjects in the control group had higher BMI and were older. The proportion of subjects taking medicines was the same for both groups, but type of medication diverged. In the intervention group, one subject used antihistamines, another paracetamol and a third contraceptives and metformin. One subject in the control group had cardiac medication and two subjects used antidepressants. Results for demographic data are shown in Table 1.

**Table 1:** Demographics and baseline variables

Variable	Intervention group (*n* = 14)	Control group (*n* = 12)	*p* value
Gender			
Female	1 (7.1%)	0 (0.0%)	
Male	13 (92.9%)	12 (100.0%)	
Age	29.9 (8.5) 27.5 (21.0; 51.0) *n* = 14	45.1 (10.1) 47.5 (29.0; 58.0) *n* = 12	< 0.01
BMI	24.9 (1.9) 24.7 (21.3; 28.7) *n* = 14	26.9 (2.8) 27.3 (23.0; 32.6) *n* = 12	0.04
Medication			
No medication	11 (78.6%)	9 (75.0%)	
Acetylsalicylic acid, Enalapril, Simvastatin and Esomeprazole	0 (0.0%)	1 (8.3%)	
Antihistamine	1 (7.1%)	0 (0.0%)	
Paracetamol	1 (7.1%)	0 (0.0%)	
SSRI	0 (0.0%)	2 (16.7%)	
Contraceptives and Metformin	1 (7.1%)	0 (0.0%)	

For categorical variables *n* (%) is presented

For continuous variables Mean (Standard deviation) / Median (Min; Max) / *n* = is presented

For comparison between groups Fisher’s Non-Parametric Permutation test was used

### GFAp

**Fig. 1:**
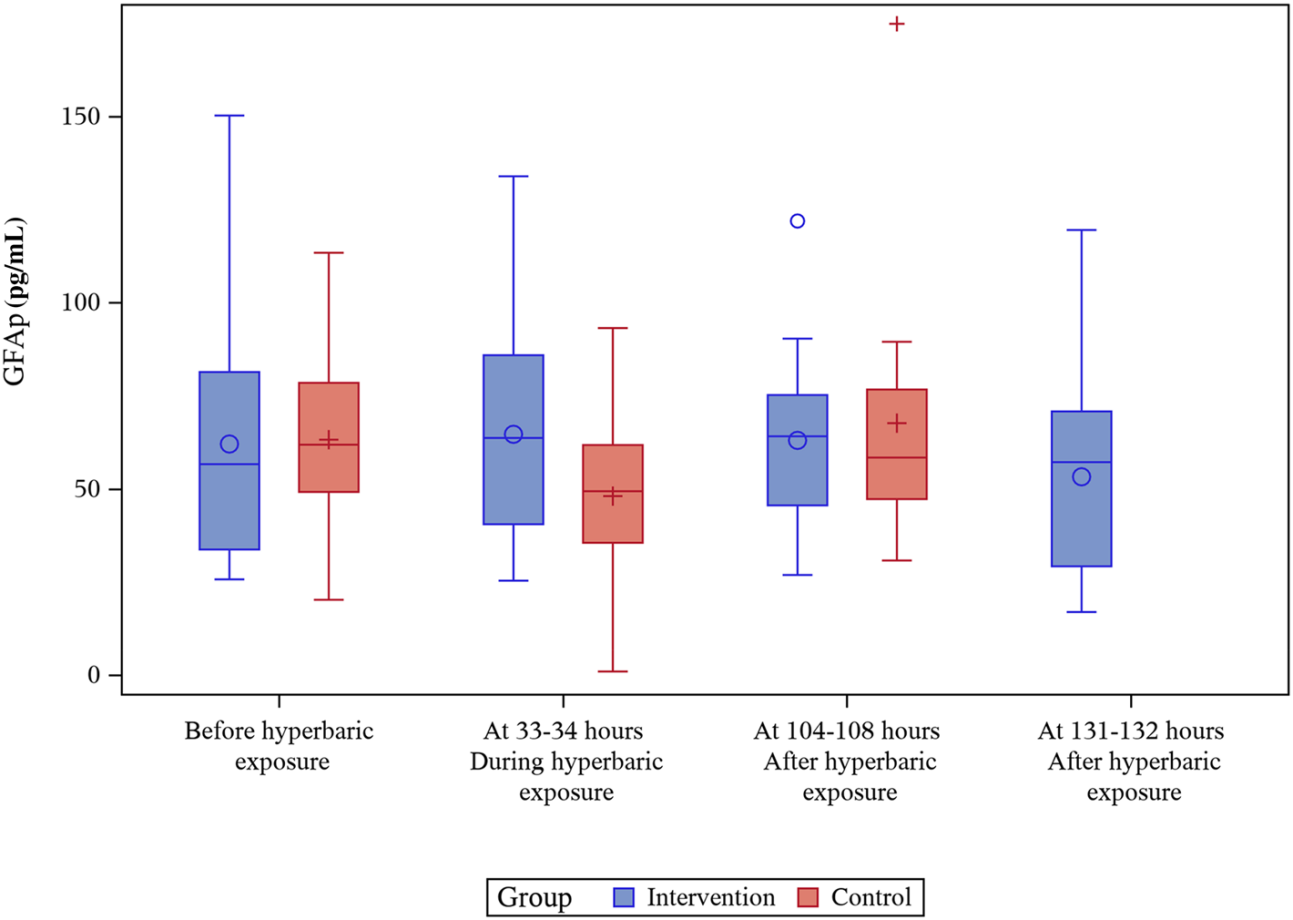
GFAp levels (pg/mL) before, during and after hyperbaric exposure

There was no difference between the groups in GFAp concentration at baseline. In the intervention group, there were no statistically significant changes in the concentration of GFAp at any point. GFAp concentration in the control group was significantly reduced in samples collected at the same time as the hyperbaric exposure was ongoing (mean/median, − 15.1/ − 8.9 pg·mL^−1^, *p* < 0.01). As a consequence, the differences in absolute change between the groups were statistically significant at this point (17.7 pg·mL^−1^, *p* = 0.02). GFAp concentration then increased in the control group, and after hyperbaric exposure, there was no difference between the groups. Results for changes in GFAp are shown in Tables 2, 3 and Fig. 1.

**Table 2:** Changes in GFAp, NFL and tau – comparisons within groups

		Intervention group (*n* = 14)			Control group (*n* = 12)		
		Absolute value			Absolute value		
**Sample 1** **Before exposure**	GFAP (pg/mL)	62.1 (33.0) 56.7 (25.7; 150.2)			63.2 (24.8) 61.9 (20.3; 113.4)		
	NfL (pg/mL)	8.29 (4.86) 6.22 (4.2; 19.01)			8.69 (5.02) 7.18 (4.12; 21.13)		
	Tau (pg/mL)	0.25 (0.23) 0.20 (0.01; 0.88)			0.34 (0.62) 0.16 (0.01; 2.28)		

		Absolute value	Absolute change compared to baseline	*p* value within group	Absolute value	Absolute change compared to baseline	*p* value within group
**Sample 2** **At 33–34 h of hyperbaric exposure**	GFAP (pg/mL)	64.7 (30.4) 63.7 (25.4; 133.9)	2.60 (17.50) 4.76 (− 33.61; 31.38) (-6.44; 11.12)	0.58	48.1 (23.7) 49.4 (1; 93.2)	− 15.1 (18.8) − 8.9 (− 62.2; 3.8) (− 26.1; -6.2)	< 0.01
	NfL (pg/mL)	9.30 (4.69) 7.58 (4.31; 20.47)	1.01 (1.80) 1.24 (− 3.57; 4.42) (0.05; 1.88)	0.06	7.36 (3.16) 7.8 (1; 13.07)	− 1.33 (3.68) 0.15 (-11.98; 1.16) (− 3.60; 0.26)	0.25
	Tau (pg/mL)	0.365 (0.35) 0.26 (0.01; 1.12)	0.11 (0.34) 0.01 (− 0.19; 1.11) (− 0.03; 0.30)	0.26	0.20 (0.21) 0.16 (0.01; 0.75)	− 0.14 (0.66) 0.02 (− 2.16; 0.39) (− 0.54; 0.11)	0.81

		Absolute value	Absolute change compared to baseline	*p* value within group	Absolute value	Absolute change compared to baseline	*p* value within group
**Sample 3** **At 104–108 h** **After hyperbaric exposure**	GFAP (pg/mL)	63.0 (25.3) 64.1 (26.9; 121.9)	0.97 (17.81) − 5.80 (− 28.3; 32.28) (− 7.96; 9.75)	0.84	67.7 (38.3) 58.4 (30.8; 174.8)	4.44 (22.76) 1.34 (− 21.46; 61.44) (− 6.86; 17.98)	0.56
	NfL (pg/mL)	8.79 (4.21) 6.73 (4.38; 18.14)	0.49 (1.46) 0.914 (− 2.40; 2.47) (− 0.280; 1.18)	0.22	7.57 (3.37) 6.87 (3.52; 16.16)	− 1.12 (3.33) − 0.17 (− 11.44; 0.92) (− 3.16; 0.13)	0.22
	Tau (pg/mL)	0.23 (0.22) 0.10 (0.01; 0.79)	− 0.02 (0.29) − 0.05 (− 0.50; 0.78) (− 0.16; 0.13)	0.77	0.15 (0.06) 0.16 (0.01; 0.25)	− 0.18 (0.62) 0 (− 2.12; 0.14) (− 0.56; 0.03)	0.41

		Absolute value	Absolute change compared to baseline	*p* value within group			
**Sample 4** **At 131–132 h** **After hyperbaric exposure**	GFAP (pg/mL)	53.3 (28.0) 57.2 (17; 119.5)	− 8.80 (23.76) − 7.11 (− 66.88; 35.75) (− 21.48; 2.66)	0.19			
	NfL (pg/mL)	7.96 (3.66) 6.78 (4.22; 16.97)	−0.33 (1.87) 0.05 (− 4.19; 2.7) (− 1.31; 0.57)	0.52			
	Tau (pg/mL)	0.34 (0.32) 0.23 (0.01; 1.22)	0.09 (0.35) 0 (− 0.26; 1.21) (− 0.06; 0.28)	0.50			

Mean (Standard deviation) / Median (Min; Max) / (Bootstrapped (10,000 replicates) 95% Confidence interval for mean) / are presented. For comparison of changes within groups the Fisher’s Non-Parametric Permutation test for matched pairs was used

**Table 3:** Changes in GFAp, NfL and tau – comparison between groups

		Intervention group (*n* = 14)	Control group (*n* = 12)		
**Sample** **1** **Before exposure**		Absolute value	Absolute value		*p* value
	GFAP (pg/mL)	62.1 (33.0) 56.7 (25.7; 150.2)	63.2 (24.8) 61.9 (20.3; 113.4)		0.92
	NfL (pg/mL)	8.29 (4.86) 6.22 (4.2; 19.01)	8.69 (5.02) 7.18 (4.12; 21.13)		0.84
	Tau (pg/mL)	0.25 (0.23) 0.20 (0.01; 0.88)	0.34 (0.62) 0.16 (0.01; 2.28)		0.83

	Variable	Absolute change	Absolute change	Absolute difference in mean change between exposed and controls	*p* value
**Sample 2** **At 33–34 h of hyperbaric exposure**	GFAP (pg/mL)	2.60 (17.50) 4.76 (− 33.61; 31.38) (− 6.44; 11.12)	− 15.1 (18.8) − 8.9 (− 62.2; 3.8) (− 26.1; − 6.2)	17.7 (4.6; 31.8)	0.02
	NfL (pg/mL)	1.01 (1.80) 1.24 (− 3.57; 4.42) (0.05; 1.88)	− 1.33 (3.68) 0.15 (− 11.98; 1.16) (− 3.60; 0.26)	2.34 (0.44; 4.76)	0.02
	Tau (pg/mL)	0.11 (0.34) 0.01 (− 0.19; 1.11) (− 0.03; 0.30)	− 0.14 (0.66) 0.02 (− 2.16; 0.39) (− 0.54; 0.11)	0.25 (− 0.07; 0.72)	0.27

	Variable	Absolute change	Absolute change	Absolute difference in mean change between exposed and controls	*p* value
**Sample 3** **At 104–108 h** **After hyperbaric exposure**	GFAP (pg/mL)	0.97 (17.81) − 5.80 (− 28.3; 32.28) (− 7.68; 10.08)	4.44 (22.76) 1.34 (− 21.46; 61.44) (-6.55; 18.01)	− 3.47 (− 19.21; 11.63)	0.68
	NfL (pg/mL)	0.49 (1.46) 0.91 (− 2.40; 2.47) (− 0.28; 1.18)	− 1.12 (3.33) -0.17 (-11.44; 0.92) (− 3.14; 0.13)	1.62 (− 0.00; 3.91)	0.07
	Tau (pg/mL)	− 0.02 (0.29) − 0.05 (− 0.50; 0.78) (− 0.16; 0.13)	− 0.18 (0.62) 0 (− 2.12; 0.14) (− 0.55; 0.03)	0.16 (− 0.13; 0.59)	0.52

Mean (Standard deviation) / Median (Min; Max) / (Bootstrapped (10,000 replicates) 95% Confidence interval for mean) are presented. Calculation of confidence interval for continuous variables (absolute change) is based on bootstrapping of 1000 replicates picking the 2.5 and 97.5 percentiles of the 10,000 mean differences as confidence interval. For difference in change between groups Mean (95% CI) is presented. For comparison between groups the Fisher’s Non-Parametric Permutation Test was used

### NfL

**Fig. 2:**
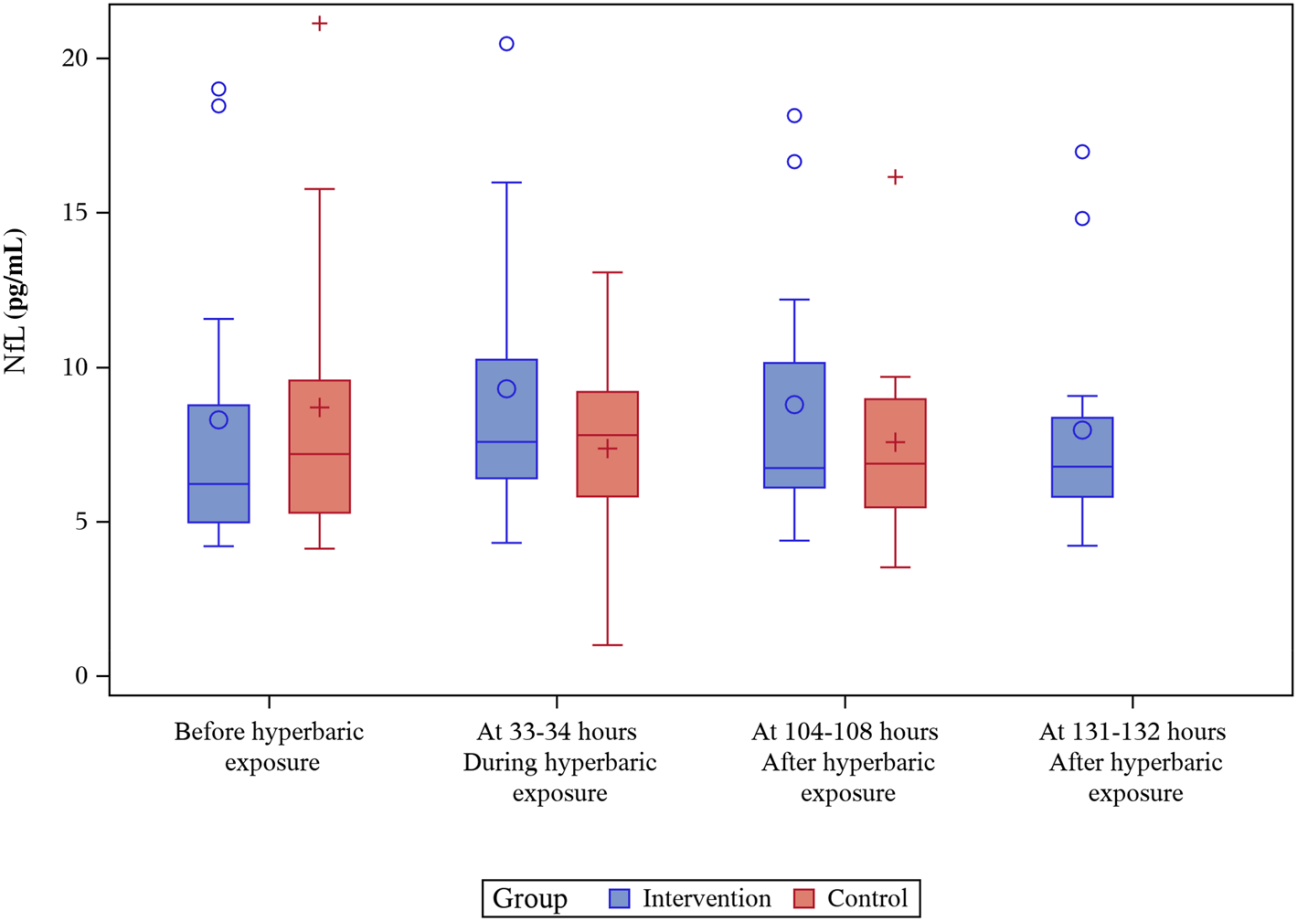
NfL levels (pg/mL) before, during and after hyperbaric exposure

There was no difference between the groups in NfL concentration at baseline. In the intervention group, an increase in NfL concentration was seen during hyperbaric exposure (mean/median 1.01/1.24 pg·mL^−1^, *p* = 0.06), after which NfL concentrations decreased. Concentration of NfL did not change significantly in the control group but the observed mean concentration was decreased. The difference in absolute NfL concentration changes between the groups was statistically significant during hyperbaric exposure of the intervention group (2.34 pg·mL^−1^, *p* = 0.02) but not when decompression had ended (1.62 pg·mL^−1^, *p* = 0.07). There were no significant changes in the intervention group. Results for changes in NfL are shown in Tables 2, 3 and Fig. 2.

### Tau

**Fig. 3:**
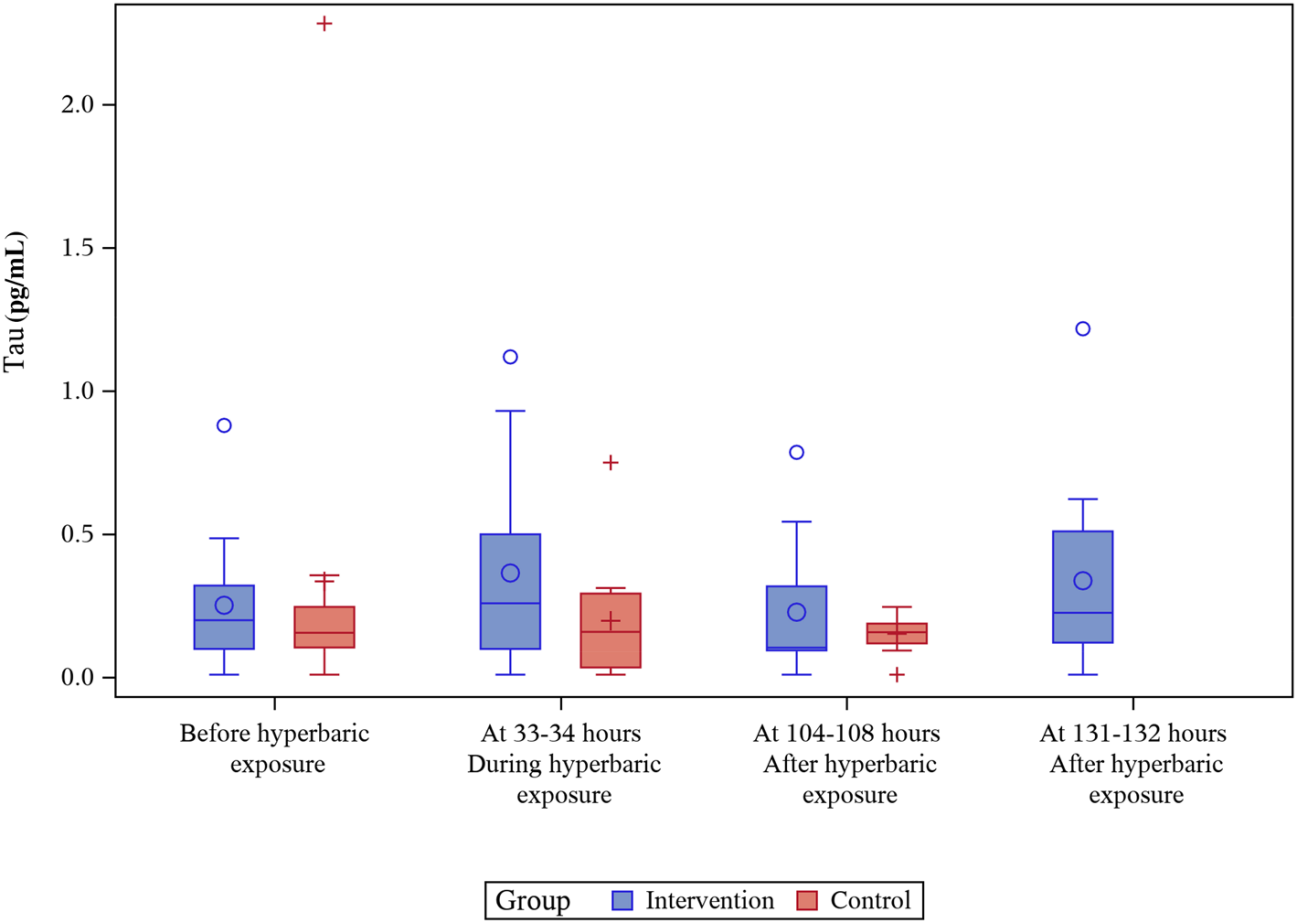
Tau levels (pg/mL) before, during and after hyperbaric exposure

There was no difference between the groups in tau concentration at baseline. Variations in mean and median tau concentrations during the study did not reach statistical significance in either group. Results for changes in tau are shown in Tables 2, 3 and Fig. 3.

### Haematocrit and haemoglobin

Directly after hyperbaric exposure, Hct had increased significantly (mean/median 2.3%/1.5%, *p* = 0.02) in the intervention group, whereas an increase in mean Hb that did not reach statistical significance was seen (4.9 g/L, *p* = 0.06). Hct and Hb were not assessed in the control group. Results for changes in Hct and Hb are shown in Table 4.

**Table 4:** Changes in Haemoglobin, Haematocrit and Albumin – comparisons within and between groups

Sample	Variable	Intervention group (*n* = 14)		Control group (*n* = 12)		*p* value between groups
		Absolute value	*p* value within group	Absolute value	*p* value within group	
**Sample 1** **Before exposure**	Albumin g/L	46.84 (3.66) 46.25 (41.7; 54.2) (44.72; 48.95) *n* = 14		47.62 (2.86) 47.25 (43.90; 51.90) (45.57; 49.67) *n* = 10		0.41
	Haemoglobin g/L	158.00 (13.06) 161.50 (136.0; 190.0) (150.46; 165.54) *n* = 14				
	Haematocrit (%)	45.61 (2.76) 46.00 (40.0; 49.0) (43.95; 47.28) *n* = 13				
**Sample 2** **At 33–34 h of hyperbaric exposure**	Albumin g/L	45.78 (3.97) 46.65 (40.0; 53.1) (43.26; 48.31) *n* = 12	0.53	44.88 (2.942) 46.30 (39.10; 49.20) (42.91; 46.86) *n* = 11	0.02	0.39
**Sample 3** **At 104–108 h** **After hyperbaric exposure**	Albumin g/L	46.69 (3.81) 46.60 (40.6; 54.3) (44.39; 48.99) *n* = 13	0.81	45.79 (2.59) 46.20 (39.40; 48.50) (44.05; 47.53) *n* = 11	0.19	0.42
	Haemoglobin g/L	162.86 (7.62) 161.50 (150.0; 180.0) (158.46; 167.26) *n* = 14	0.06			
	Haematocrit (%)	47.86 (2.25) 47.50 (44.0; 53.0) (46.56; 49.16) *n* = 14	0.02			
**Sample 4** **At 131–132 h After hyperbaric exposure**	Albumin g/L	46.02 (3.19) 45.20 (42.1; 52.7) (44.18; 47.87) *n* = 14	0.12			

For continuous variables Mean (Standard deviation) / Median (Min; Max) / (95% Confidence interval for mean) / *n* = are presented

Within each group, absolute values for haemoglobin and haematocrit were compared to baseline using Wilcoxon signed-rank test. For comparison of albumin values between groups Mann–Whitney test was used

### Albumin

There was no difference in albumin concentration between the groups at baseline. In the intervention group, there were no significant changes in albumin concentration, neither during nor after hyperbaric exposure. Albumin concentration had decreased significantly in the control group when sampled concurrently with the hyperbaric exposure (mean/median − 2.74 g/L/ − 0.95 g/L, *p* = 0.02). There was, however, no statistically significant difference between the two groups at any point. Results for changes in albumin are shown in Table 4.

### Venous gas emboli

No VGE were detectable using precordial Doppler ultrasound.

## Conclusion

In the present study, hyperbaric exposure to 401 kPa during 36 h was not associated with statistically significant increases in GFAp, NfL or tau concentrations in serum. Oxygen partial pressure never exceeded 50 kPa during exposure and the decompression regimen employed was selected to be conservative, without any need for oxygen breathing during the ascent (Gennser 2019). The nitrogen partial pressure reached levels that would be expected to mildly affect the CNS. By keeping known pertinent factors at or below levels where they would be expected to cause harmful effects, study subjects were kept as safe as possible although the environmental conditions were sufficient to induce neurological stress.

Tau, GFAp, and NfL are established as markers of brain injury after trauma and cerebral apoplexia, but they have also been reported to increase in conjunction with far less traumatising neurological stressors, such as protracted apnea, deep diving, and uneventful anaesthesia. Furthermore, both GFAp and tau are claimed to be secreted in response to increased neuronal activity and stress. It is plausible that the CNS is affected, prior to clinical symptoms becoming evident, by increased ambient pressures, increased nitrogen and oxygen partial pressures, or a combination of these and, hence, these potentially neurological harmful exposures could result in elevated levels of tau, GFAp and NfL.

During hyperbaric exposure, an increase in NfL concentration was seen in the intervention group, but it did not reach statistical significance. At the same time, mean, but not median, NfL concentration decreased in the control group, which resulted in a statistically significant difference in NfL absolute change between the groups at that point. Like NfL, GFAp concentration decreased in samples collected from the control group, while the intervention group was pressurized, causing a statistically significant difference in absolute GFAp change between the groups.

A strength of the study was that samples were collected during subject exposure to 401 kPa. It was thereby possible to obtain analyses devoid of VGE, at a point when the subjects were influenced by hydrostatic and gas pressures alone. Decompression of samples collected from the intervention group during hyperbaric exposure might have generated VGE in the test tubes, but the size and structure of GFAp, NfL, and tau molecules make it most unlikely that they could have been degraded or deformed by gas bubbles. There was no neuronal tissue in the venous blood samples which precluded further increase in GFAp, NfL, and tau concentration in the test tubes during decompression.

After completed decompression, no VGE was found in any of the subjects. Although there is a known variability in VGE formation after diving, the lack of VGE in our study most likely could be explained by a conservative decompression regimen. In the absence of observed VGE, changes in GFAp, NfL and tau would likely have been caused by increased ambient pressure alone.

The results from this study contrasted with results from a small pilot study on 10 divers, where tau concentration had increased significantly, but NfL and GFAp concentrations were unchanged after repeated diving during four days to at most between 52 and 90 m (Rosén 2019). However, hyperbaric exposure to 401 kPa followed by slow decompression is a qualitatively different exposure compared to repeated and deep diving and the results could be consistent with each other. In the diving study, subjects used trimix, a breathing gas containing oxygen, helium and nitrogen, and partial pressures of oxygen were kept at 130 kPa, whereas in the present study, the breathing gases were oxygen and nitrogen with the partial pressure of oxygen never exceeding 50 kPa. Factors, such as helium or oxygen partial pressures, might have caused the increased tau value seen in the aforementioned diving study, in which the decompression stress was also greater. Contrary to the present study, in the diving study, Doppler detectable VGE was present after most of the dives. Additionally, immersion in water causes redistribution of blood flow, which could potentially change cerebral perfusion and cerebral gas pressures, and thereby affect the release of GFAp, NfL, and tau into the blood. It is possible that the hyperbaric exposure and decompression regimen employed in this study was not sufficiently challenging to affect the CNS in such a way that serum GFAp, NfL or tau concentrations were increased, given that the oxygen partial pressure was kept below what is considered a toxic level, and the decompression stress appeared mild.

Samples taken during and after hyperbaric exposure were collected at approximately the same time of day, which excludes diurnal variation as a cause. Diurnal variation of Hct and albumin has been reported to be < 3% (Sennels 2011, Andersen 2015). It is also unlikely that the decrease in GFAp and NfL in the control group represented an impaired circadian rhythm, as this phenomenon was seen neither in the intervention group nor later. It seems implausible that the lower GFAp and NfL concentrations could be explained by age or BMI but unknown confounding factors and selection bias may have affected the results. Measurement error is improbable as the internal quality control samples gave the expected values.

It is possible that level of hydration did influence the measured concentrations of GFAp, NfL, and tau. Hb, Hct (Matomäki 2018) and albumin (Miller 2019) have been used to assess hydration status. Prolonged exposure to increased ambient pressure may result in decreased Hb levels (Hofsø 2005, Luczynski 2019) that return to prior levels after cessation of exposure, which could influence estimation of hydration status. The study was conducted at a naval base in Sweden. Hospital laboratory facilities were too far away to make centrifugal measurement of Hct possible. The i-STAT blood analyser was chosen to measure Hb and Hct because it was portable. The important fact that i-STAT determined Hct conductometrically was, unfortunately, not recognised until the study was actually carried out. Consequently, all Hct samples taken during hyperbaric exposure had to be discarded, as potential VGE induced by decompression of the samples interfered with the results of the measured conductivity. This error could have been avoided with better planning and is an obvious shortcoming of the study that should be avoided in the future.

Mean Hct and Hb values obtained in the intervention group had increased after hyperbaric exposure but the change was only significant for Hct. Valid albumin measurements from both studied groups were available before, during and after hyperbaric exposure. There were never any significant differences in albumin values between the groups, but the spurious decrease in albumin in the control group at the time when the intervention group was exposed to hyperbaric pressure makes a relative dehydration in the intervention group possible. Measured albumin values are by many hospital laboratories reported to be 10–15% lower if taken during bed rest. During the present study, subjects in the intervention group were mainly inactive in a hyperbaric chamber while subjects in the control group moved as usual. This may have biased the albumin results giving comparatively lower albumin results in the intervention group, which would point to a larger actual difference in albumin values between the groups than measured. Due to the lack of Hct measurements during hyperbaric exposure assessment of hydration was less precise, but the small increase in Hct after exposure and the unchanged albumin values among exposed individuals make it improbable that significant dehydration confounded the results. Differences between the groups during hyperbaric exposure were predominantly due to the decrease in GFAp and NfL in the control group. If dehydration was present among individuals in the intervention group, a significant increase in GFAp, NfL, and tau would have been expected, which was not the case.

There were a few GFAp, NfL, and tau values, most notably in the intervention group, that differed considerably from the median. It is unclear if they were caused by measurement errors or physiological diversity. These values represented 1–2 individuals in the control group and 1–3 individuals in the intervention group, respectively, depending on protein analysed. As a result, the distribution of results was partly skewed but as both mean and median values were reported and a non-parametric statistical technique used, disproportionate weight to outliers was averted.

The study was carried out during naval training. Hence, the number of eligible study subjects was limited and the hyperbaric regimen predetermined. The small number of subjects was a weakness of the study. There is a possibility that an effect did exist and that a larger study would have yielded statistically significant results.

In conclusion, hyperbaric exposure to 401 kPa for 36 h followed by slow decompression over 70 h was not associated with statistically significant increases in GFAp, NfL or tau concentrations. Diurnal variation, changes in albumin levels or hydration were considered unlikely to have confounded the results but the lack of information about Hct made assessement of hydration status during hyperbaric exposure less precise. A larger study with an appropriate control group and measurement of Hct levels during hyperbaric exposure is needed to validate these results. Future studies on divers both with and without DCS would be desirable to establish the role of GFAp, NfL, and tau in hyperbaric research. Nevertheless, in view of the presented results, saturation exposure to 401 kPa seems to be a procedure not harmful to the CNS.

## Abbreviations

BMI: Body mass index
CNS: Central nervous system
CV: Coefficients of variation
DCS: Decompression sickness
GFAp: Glial fibrillary acidic protein
Hb: Haemoglobin
Hct: Haematocrit
kPa: Kilopascal
msw: Meters of seawater
NfL: Neurofilament light
SwAF: Swedish armed forces
tau: Protein tau
UCH-L1: Ubiquitin carboxy-terminal hydrolase L-1
VGE: Venous gas emboli
QC: Quality control

## References

[CR1] Andersen IB, Brasen CL, Christensen H (2015). Standardised testing time prior to blood sampling and diurnal variation associated with risk of patient misclassification: results from selected biochemical components. PLoS ONE.

[CR2] Balestra C, Germonpré P (2016). Correlation between patent foramen ovale, cerebral “lesions” and neuropsychometric testing in experienced sport divers: does diving damage the brain?. Front Psychol.

[CR3] Bast-Petrersen R, Skare Ø, Nordby K-C, Skogstad M (2015). A twelve-year longditudinal study of neuropsychological function in non-saturation professional divers. Int Arch Occup Environ Haelth.

[CR4] Bennet P, Rostain JC, Brubakk AO, Neumann TS (2003). Inert gas narcosis. Bennet and Elliot’s physiology and medicine of diving.

[CR5] Brenner M (2014). Role of GFAP in CNS injuries. Neurosci lett.

[CR6] Cordes P, Keil R, Bartsch T (2000). Neurologic outcome of compressed-air diving. Neurology.

[CR7] Edmonds C, Bennet M, Lippmann J, Mitchell SJ (2016). Oxygen toxicity. Diving and subaquatic medicine.

[CR8] Edmonds C, Bennet M, Lippmann J, Mitchell SJ (2016). Carbon dioxide toxicity. Diving and subaquatic medicine.

[CR9] Eftedal OS, Lydersen S, Brubakk AO (2007). The relationship between gas bubbles and adverse effects of decompression after air dives. Undersea Hyperb Med.

[CR10] Evered L, Silbert B, Scott DA, Zetterberg H, Blennow K (2018). Association of changes in plasma neurofilament light and tau levels with anesthesia and surgery. JAMANeurol.

[CR11] Femenia T, Gimenez-Cassina A, Codeluppi S (2018). Disrupted neuroglial metabolic coupling after peripheral surgery. J Neurosci.

[CR12] Foerch C, Niessner M, Back T (2012). Diagnostic accuracy of plasma glial fibrillary acidic protein for differentiating intracerebral haemorrhage and cerebral ischemia in patients with symptoms of acute stroke. Clin Chem.

[CR13] Francis TJR, Mitchell SJ, Brubakk AO, Neumann TS (2003). Pathophysiology of decompression sickness. Bennet and Elliot’s physiology and medicine of diving.

[CR14] Gennser M, Grönkvist M (2019). Utprovning av mättnadsdekompressionstabell från grund luft- och nitroxmättnad.

[CR15] Germonpré P, Balestra C (2017). Preconditioning to reduce decompression stress in scuba divers. Aerosp Med Hum Perform.

[CR16] Gill J, Latour L, Diaz-Arrastia R (2018). Glial fibrillary acidic protein elevations relate to neuroimaging abnormalities after mild TBI. Neurology.

[CR17] Gren M, Shahim P, Lautner R, Wilson DH, Andreasson U (2016). Blood biomarkers indicate mild neuroaxonal injury and increased amyloid beta production after transient hypoxia during breath-hold diving. Brain Inj.

[CR18] Grønning M, Aarli JA (2011). Neurological effects of deep diving. J Neurol Sci.

[CR19] Hamilton RW (1989). Tolerating exposures to high oxygen levels. Repex and other methods. Mar Tech Soc J.

[CR20] Hemelryck W, Germonpre P, Papadopoulou V, Rozloznik M, Balestra C (2014). Long term effects of recreational SCUBA diving on higher cognitive function. Scand J Med Sci Sports.

[CR21] Hofsø D, Ulvik RJ, Segadal K, Hope A, Thorsen E (2005). Changes in erythropoietin and haemoglobin concentrations in response to saturation diving. Eur J Appl Physiol.

[CR22] Kahlil M, Teunissen CE, Otto M, Piehl F, Sormani MP (2018). Neurofilaments as biomarkers in neurological disorders. Neurology.

[CR23] Le Péchon JC, Gourdon G (2010). Compressed-air work is entering the field of high pressures. Undersea Hyperb Med.

[CR24] Luczynski D, Lautridou J, Hjelde a, Monnoyer R, Eftedal I, (2019). Hemoglobin during and following a 4-week commercial saturation dive to 200 m. Front Physiol.

[CR25] Matomäki P, Kainulainen H, Kyröläinen H (2018). Corrected whole blood biomarkers – the equation of Dill and Costill revisited. Physiol Reports.

[CR26] Mattson N, Zetterberg H, Nielsen N, Blennow K, Dankiewicz J (2017). Serum tau and neurological outcome in cardiac arrest. Ann Neurol.

[CR27] Miller GD, Teramoto M, Smeal SJ, Cushman D, Eichner D (2019). Assessing serum albumin concentration following exercise-induced fluid shifts in the context of the athlete biological passport. Drug test Anal.

[CR28] Neselius S, Brisby H, Theodorsson A, Blennow K, Zetterberg H, Marcusson J (2012). CSF-biomarkers in Olympic boxing: Diagnosis and effects of repetitive head trauma. PLoS ONE.

[CR29] Nishi RY, Brubakk AO, Eftedal OS, Brubakk AO, Neumann TS (2003). Bubble detection. Bennet and Elliot’s physiology and medicine of diving.

[CR30] Papadopoulou V, Germonpré P, Cosgrove D (2018). Variability in circulating gas emboli after a same scuba dive exposure. Eur Appl Physiol.

[CR31] Powell M (2014). Saturation diving. Deco for divers.

[CR32] Rosén A, Oscarsson N, Kvarnström A, Gennser M, Sandström G (2019). Serum tau concentration after diving – an observational pilot study. Diving Hyperb Med.

[CR33] Sato C, Barthélemy NR, Mawuenyega KG (2018). Tau kinetics in neurons and the human nervous system. Neuron.

[CR34] Sennels HP, Jørgensen HL, Hansen ALS, Goetze JP, Fahrenkrug J (2011). Diurnal variation of hematology parameters in healthy young males: the Bispebjerg study of diurnal variations. Scand J Clin Lab Invest.

[CR35] Shahim P, Tegner Y, Wilson DH, Randall J, Skillbäck T (2014). Blood biomarkers for brain injury in concussed professional ice hockey players. JAMA Neurol.

[CR36] Shahim P, Arnell P, Kvarnström A, Rosén A, Bremell D (2015). Cerebrospinal fluid markers of central nervous system injury in decompression illness - a case-controlled pilot study. Diving Hyperb Med.

[CR37] Shahim P, Gren M, Liman V, Andreasson U, Norgren N (2016). Serum neurofilament light protein predicts clinical outcome in traumatic brain injury. Sci Rep.

[CR38] Shahim P, Tegner Y, Marklund N, Blennow K, Zetterberg H (2018). Neurofilament light and tau as blood biomarkers for sports-related concussion. Neurology.

[CR39] Slosman DO, de Ribaupierre S, Chichero C (2004). Negative neurofunctional effects of frequency, depth and environment in recreational SCUBA diving: the Geneva “memory dive” study. Br J Sports Med.

[CR40] Taylor CL, Macdiarmid JI, Ross JAS (2006). Objective neuropsychological test performance of professional divers reporting a subjective complaint of “forgetfulness or loss of concentration”. Scand J Work Environ Health.

[CR41] Todnem K, Nyland H, Kambestad BK, Aarli JA (1990). Influence of occupational diving upon the nervous system: an epidemiological study. Br J Ind Med.

[CR42] Todnem K, Nyland H, Skeidsvoll H, Svihus R, Rinck P (1991). Neurological long term consequences of deep diving. Br J Ind Med.

[CR43] Wang YF, Hatton GI (2009). Astrocytic plasticity and patterned oxycontin neuronal activity: dynamic interactions. J Neurosci.

[CR44] Zetterberg H, Blennow K (2016). Fluid biomarkers for mild traumatic brain injury and related conditions. Nat Rev Neurol.

[CR45] Zetterberg H, Hietala A, Jonsson M (2006). Neurochemical aftermath of amateur boxing. Arch Neurol.

